# Food parenting practices for 5 to 12 year old children: a concept map analysis of parenting and nutrition experts input

**DOI:** 10.1186/s12966-017-0572-1

**Published:** 2017-09-11

**Authors:** Teresia M. O’Connor, Louise C. Mâsse, Andrew W. Tu, Allison W. Watts, Sheryl O. Hughes, Mark R. Beauchamp, Tom Baranowski, Truc Pham, Jerica M. Berge, Barbara Fiese, Rebecca Golley, Melanie Hingle, Stef P. J. Kremers, Kyung E. Rhee, Helen Skouteris, Amber Vaughn

**Affiliations:** 1 USDA/ARS Children’s Nutrition Research Center, Baylor College of Medicine, 1100 Bates St, Houston, TX USA; 2 BC Children’s Hospital Research Institute, School of Population and Public Health, University of British Columbia, Vancouver, BC Canada; 3 Child & Family Research Institute, School of Population and Public Health, University of British Columbia, Vancouver, BC Canada; 4 Division of Epidemiology and Community Health, School of Public Health, University of Minnesota, Minneapolis, MN USA; 5 School of Kinesiology, University of British Columbia, Vancouver, BC Canada; 6 Department of Family Medicine and Community Health, University of Minnesota Medical School, Minnesota, MN USA; 7 Family Resilience Center, Department of Human Development and Family Studies, University of Illinois at Urbana-Champaign, Urbana, IL USA; 8 Sansom Institute for Health Research (PHRC), School of Pharmacy and Medical Sciences, University of South Australia, Adelaide, Australia; 9 Department of Nutritional Sciences, College of Agriculture and Life Sciences, University of Arizona, Tucson, AZ USA; 10 NUTRIM School of Nutrition and Translational Research in Metabolism, Maastricht University Medical Center, Maastricht, The Netherlands; 11 Department of Pediatrics, University of California, San Diego, CA USA; 12 School of Psychology, Deakin University, Melbourne, Australia; 13 Department of Nutrition, Gillings School of Global Public health, University of North Carolina, Chapel Hill, NC USA

**Keywords:** Food, Nutrition, Child, Parenting, Parenting practices, Family, Concept mapping, Measurement

## Abstract

**Background:**

Parents are an important influence on children’s dietary intake and eating behaviors. However, the lack of a conceptual framework and inconsistent assessment of food parenting practices limits our understanding of which food parenting practices are most influential on children. The aim of this study was to develop a food parenting practice conceptual framework using systematic approaches of literature reviews and expert input.

**Method:**

A previously completed systematic review of food parenting practice instruments and a qualitative study of parents informed the development of a food parenting practice item bank consisting of 3632 food parenting practice items. The original item bank was further reduced to 110 key food parenting concepts using binning and winnowing techniques. A panel of 32 experts in parenting and nutrition were invited to sort the food parenting practice concepts into categories that reflected their perceptions of a food parenting practice conceptual framework. Multi-dimensional scaling produced a point map of the sorted concepts and hierarchical cluster analysis identified potential solutions. Subjective modifications were used to identify two potential solutions, with additional feedback from the expert panel requested.

**Results:**

The experts came from 8 countries and 25 participated in the sorting and 23 provided additional feedback. A parsimonious and a comprehensive concept map were developed based on the clustering of the food parenting practice constructs. The parsimonious concept map contained 7 constructs, while the comprehensive concept map contained 17 constructs and was informed by a previously published content map for food parenting practices. Most of the experts (52%) preferred the comprehensive concept map, while 35% preferred to present both solutions.

**Conclusion:**

The comprehensive food parenting practice conceptual map will provide the basis for developing a calibrated Item Response Modeling (IRM) item bank that can be used with computerized adaptive testing. Such an item bank will allow for more consistency in measuring food parenting practices across studies to better assess the impact of food parenting practices on child outcomes and the effect of interventions that target parents as agents of change.

**Electronic supplementary material:**

The online version of this article (doi:10.1186/s12966-017-0572-1) contains supplementary material, which is available to authorized users.

## Background

Most children’s eating patterns and behaviors are shaped by family influences and ultimately can have an important impact on their weight status [1–3]. Research designed to better understand how parents influence their children’s eating has grown over the past two decades and has resulted in over 75 published articles related to the development of unique food parenting instruments [4]. Most of this work has focused on food parenting practices, or the specific goal-directed parent actions designed to influence children’s eating behaviors or dietary intake [5]. With this growing number of available instruments, there is little consensus on how to measure food parenting practices, including which instrument to use and how food parenting constructs relate to or correlate with each other. This significantly limits our ability to evaluate the relationships between various food parenting constructs and children’s intake or weight status; or compare findings across studies [6, 7].

Proposed ways to advance or improve the measurement of food parenting practices on children’s eating behaviors and dietary intake include using direct or video observational methods [8] or employing digital technologies or simulations [6]. However, many large descriptive cross-sectional or prospective studies, or interventions will not be able to utilize such assessments due to the associated costs or burden on participants. Enhancing the ways in which behavioral and public health scientists can reliably and validly assess food parenting practices in a standard way via self-report is vital to advancing the field. One method for improving and standardizing the measurement of latent constructs measured by self-report is Item Response Modeling (IRM) of an item bank, supplemented with computerized adaptive testing [6, 9, 10]. In this approach, a bank of items that assesses the latent construct is developed and calibrated by IRM analysis. Computer adaptive testing of the calibrated item bank allows researchers to select all or a subset of the calibrated items to use, while maintaining the ability to compare the resulting score for the latent construct across studies. For a complex idea with multiple constructs, such as those that correspond to food parenting practices, a conceptual framework is needed to inform how the food parenting practice constructs are operationalized. While a content map for food parenting practices has recently been proposed [11], there is no tested consensus for how specific food parenting practice concepts or corresponding items fit within each construct of the proposed framework. To inform this process, this study aimed to develop a food parenting practice conceptual framework for parents with children 5–12 years old based on an existing systematically derived item bank of food parenting practices [4] using i) an online card sort task conducted by an international sample of experts of food parenting and feeding, followed by ii) a concept mapping analysis of the resulting grouping of food parenting concepts into constructs and a larger framework. The long-term goal of this project is to develop a calibrated IRM item bank that can be used with computerized adaptive testing and can be utilized by other researchers in the food parenting field internationally.

## Method

### Identification of expert panel

Scientific experts were recruited to help develop the conceptual framework. Experts were defined as researchers who have either a) developed nutrition-based, family interventions aimed at treating or preventing childhood obesity and/or modifying dietary behaviors; or b) studied the role of parenting and nutrition in the etiology of childhood obesity. A list of experts was created by reviewing: 1) the membership list of the International Society of Behavioral Nutrition and Physical Activity (ISBNPA); 2) the list of attendees to the 2012 pre-ISBNPA workshop focused on improving measures of physical activity and food parenting practices; 3) recent publications on food parenting practices through searches on PubMed, ERIC, PsycINFO, and ScienceDirect; and 4) asking our network of researchers for additional suggestions. In total 32 experts were identified and 25 experts from 8 countries (Australia, Canada, Finland, Japan, Mexico, Netherlands, UK, and USA) agreed to participate (78% response rate). All experts were offered an honorarium ($150) for their participation. The sorting task included participation of 28 experts, the 25 outside experts and three primary members of the research team (*TB, TMO, and SOH*) who did not conduct the statistical analysis. The protocol was approved by the Institutional Review Boards at the University of British Columbia and Baylor College of Medicine.

### Identification, reduction and sorting of food parenting practices

**Fig. 1:**
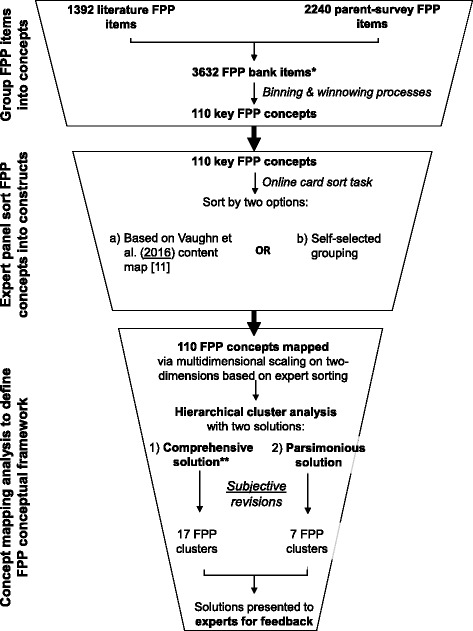
Overview of the Methods to Develop the Food Parenting Practice (FPP) Concept Map. *Based on systematic item bank and first round of binning/winnowing published by O'Connor et al. [4]. **Based on content map published by Vaughn et al. [11]

An overview of the methods of this study can be found in Fig. 1. Previous work by our group [4] systematically identified published food parenting instruments and supplemented the published items with additional items reported by parents to populate a food parenting practice item bank. Briefly, published articles containing at least one scale on parenting or caregiver behaviors related to 2 to 16 year old children’s eating, nutrition, or food intake were extracted from 1) articles identified from two recent systematic reviews; [12, 13] 2) an additional systematic review of articles published between January 2009 and March 2013 in PubMed, ERIC, PsycINFO, and ScienceDirect; [4] and 3) reviewing and back-tracing the reference of articles from steps 1 and 2. The broader age range for the review compared to the ultimate target age range of the item bank (5–12 year old children) was selected in order to capture a wide range of items. A total of 79 measures were identified consisting of 1392 items measuring food parenting practices [4]. To ensure data saturation of food parenting practices for the item bank, 135 parents who reflected the socio-economic and ethnic diversity specific to Canada and the US, were surveyed by an online polling firm (YouGovPolimetrix, USA) about food parenting practices they have used or think other parents use [4]. They contributed 2240 valid (1985 unique, after removal of duplicates) food parenting practices, many that overlapped with published items [4]. To reduce the 3632 food parenting practice items identified from the published literature and parent reports and make the sorting task manageable for the experts, the binning and winnowing process, developed by the NIH PROMIS initiative was used [9, 10]. “Binning,” or grouping similar food parenting practice items, consisted of assigning the items from the literature review and responses from the parent survey to one of 19 primary codes and a subsequent secondary code [4]. “Winnowing” or removing redundant items consisted of reviewing each bin and consolidating redundant items. The binning and winnowing process was conducted by two research members independently with all discrepancies triangulated by two other members of the research team until a consensus was reached among all four. Two rounds of binning and winnowing of the initial 1392 items found in the literature and the 2240 parent responses took place (see previously published work for first round) [4] and the final round resulted in 110 key parenting practice concepts. A food *parenting practice concept* could represent a number of food parenting practice *items*. For example, one food parenting practice concept was “I reward my child with something tasty (e.g., dessert) as a way to get him/her to eat [food]” where food could represent “healthy food”, “all his dinner”, or “fruits and vegetables”. This concept represented a total of 43 items from the published literature or statements from parent report. The food parenting practice concepts were then grouped into food parenting practice *constructs* via Expert Panel sorting.

The participating experts were invited to sort the 110 key food parenting practice concepts into meaningful groups or constructs using the web-based Concept Mapping software (Concept Systems Inc., Ithaca, NY). To take advantage of existing substantial conceptual interpretation of food parenting practices, each expert was provided a copy of the previously published Vaughn et al. 2016 content map [11] prior to sorting and instructed to a) utilize the framework to guide their sorting and/or b) to propose a different grouping of food parenting practice concepts. The published content framework grouped food parenting practices into 19 constructs stemming from three larger domains: control, structure and autonomy promotion based on the authors’ critical appraisal of the literature [11].

In addition to sorting the concepts into meaningful groups, the experts were asked to name the groups they created. They were also instructed to not group unique practices together (i.e., create a miscellaneous group of leftover practices), but instead create groups of single food parenting practice concepts if only one practice fit within the group. The sorting conducted by the experts was reviewed to ensure that each expert sorted all 110 statements and that no miscellaneous group was formed. One expert did create a miscellaneous group of 10 food parenting practice concepts. Follow up with this expert, resulted in all those concepts being sorted into existing or new categories.

### Analysis

Analysis of the sorting was conducted using non-parametric multidimensional scaling (MDS) [14]. A two-dimensional solution was used to assign each food parenting practice concept an x/y coordinate on a point map. Food parenting practice concepts that appeared spatially closer to one another on the point map were grouped by the experts closer together and therefore may represent a similar construct. Acceptable stress values for MDS analysis typically range from 0.205 to 0.365 when used to develop a conceptual framework [15], as opposed to when used in controlled psychometric evaluations, which typically necessitate lower stress values (note that the MDS stress value for our solution was 0.267 and within acceptable range) [16].

A hierarchical cluster analysis was then conducted to identify clusters of food parenting practice concepts from the MDS derived point map. Specifically, the hierarchical cluster analysis was carried out on the x/y coordinates which were obtained from the MDS analysis. The concept mapping software utilizes the Ward’s algorithm for the cluster analysis because it: 1) retains the location of the x/y coordinates in the final solution; 2) creates non overlapping constructs; and 3) merges clusters based on the distance of all individual statements instead of using the centroid of a cluster [14].

We adapted the procedure outlined by Trochim [14] to identify the appropriate number of clusters to retain in our solution. Trochim’s approach to identify the number of clusters retained in the solution is iterative but essentially starts by: 1) reviewing an initial cluster solution that is derived statistically with more clusters that would be anticipated; 2) adding more clusters one at a time until it makes no theoretical sense to combine clusters; 3) qualitatively reviewing the statistical solution to refine and fine-tune the shape of the clusters; and 4) having experts review the solution and provide further input into the analyses. As we aimed to identify two potential solutions, we refined this process for the following two solutions: 1) a parsimonious solution and 2) a solution that approximated the Vaughn et al., 2016 content map [11] (referred herein as the comprehensive solution). The parsimonious solution was identified by first evaluating the simplest cluster analysis-generated solution and determining whether adding another cluster based on the cluster analysis made conceptual sense. This process iteratively continued and stopped when it did not make sense to add further clusters. This solution was not constrained by a pre-determined conceptual framework but aimed to identify a parsimonious solution, meaning we looked for larger clusters that contained related food parenting practice concepts. The solution was then examined and subjectively modified to integrate the team’s consensus solution of the two-dimensional point map. Specifically, the content of each cluster was examined, with emphasis on food parenting practice concepts at the border of each cluster to assess whether it could better fit with another cluster, prioritizing neighboring clusters when appropriate. This iterative process continued until the final solution was obtained.

We identified the comprehensive solution by using the Vaughn et al. 2016 content map [11] to initially examine a larger than expected cluster solution. We arbitrarily started by examining the 28-cluster solution and then determined whether reducing the cluster analysis derived solution into fewer clusters made conceptual sense based on the Vaughn’s content map. We proceeded until merging could no longer be supported by the framework. Again, after we identified a statistical solution (potential number of clusters to retain), we subjectively reviewed the solution to determine an optimal solution that integrated the MDS results and the subjective evaluation of the two-dimensional point map using the same procedure described above. Three members of the research team (TO, LCM & AT) independently conducted these subjective analyses and their consensus solution was presented to the primary team of investigators (SH, MB, and TB) who provided initial feedback for modification and agreed on a solution to be presented to the Expert Group. We presented the two solutions to the Expert Group who were asked to select their preferred solution and provide feedback and suggestions on that solution. One last round of modifications to both solutions was conducted based on the expert’s feedback until consensus was reached among the authors.

## Results

### Expert sorting

The 28 participating Experts sorted the food parenting practice concepts into 3–28 categories, with a mean (standard deviation) of 18.1 (6.5) and mode of 19 food parenting practice categories. Six Experts sorted the food parenting practices concepts into 19 categories, the same number as presented by the Vaughn et al. 2016 content map [11]. Of those, there was overlap in the names of 5–19 constructs (mean 14.7, stand dev 5.8) with the content map, with only two having exactly the same structure (19/19 constructs) as the proposed content map [11]. It is not known how many elected to use the published content guide to inform their sorting. However, in reviewing the names of categories proposed by the Experts, many used at least some of the same construct names while adding to and/or deleting food constructs for their final solution.

### Expert preference for proposed solutions

**Fig. 2:**
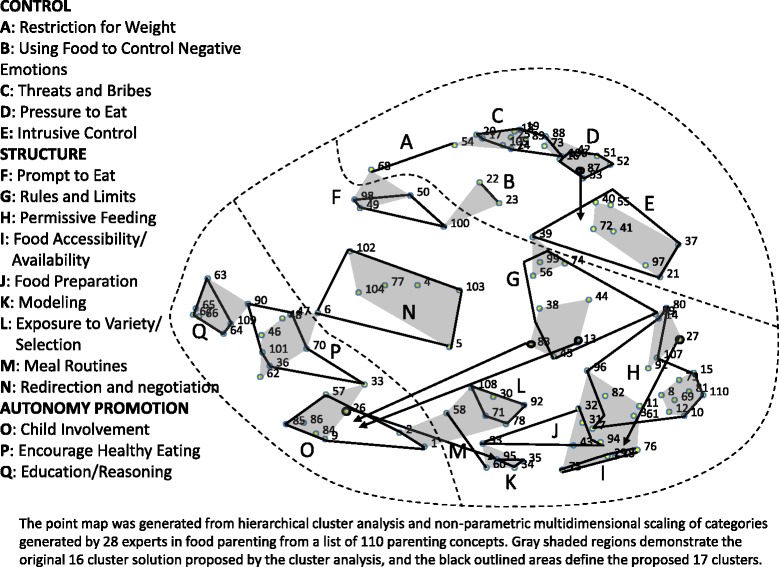
Comprehensive solution for food parenting statements subjectively grouped into clusters, informed by the hierarchical cluster analysis and a published framework (Vaughn et al. [11]).

Both the parsimonious and the comprehensive concepts map solutions were presented to the original experts who participated in the sorting task. Of the 27 eligible expert respondents (TMO was excluded because she managed the responses), 23 responded (85.2%). The comprehensive concept map informed by the published content map [11] (Fig. 2) was preferred by 52% of experts, and another 35% preferred to present both solutions. Based on these preferences, we include the comprehensive concept map informed by the published content map within this article, but have made the parsimonious solution available online in an Additional file 1.

Experts reported they preferred the comprehensive solution because it was more theoretically based and the specific differentiation of food parenting practices had promise for better informing which food parenting practices were most important in influencing child eating behaviors. The most common reason for preferring to present both solutions was that the two frameworks had the potential for serving different purposes, with the comprehensive solution being more applicable to researchers in this area and the parsimonious solution being useful for those who try to operationalize promoting these practices in obesity prevention programs or policy statements. A few experts suggested future work may be able to integrate the two models into one model, with a more parsimonious global solution and detailed “sub-factors” embedded within the parsimonious constructs.

### The Comprehensive conceptual framework of food parenting practices

The comprehensive food parenting practices concept map based on the published content map [11] resulted in an 17-cluster solution from a statistically derived 16 cluster solution (see Fig. 2 with concepts, construct names, and definitions listed in Table 1) with subjective modifications. Vaughn et al., proposed grouping food parenting practices into three larger overarching domains: Control, Structure, and Autonomy Promotion [11]. Figure 2 illustrates how the comprehensive concept map potentially supports these same three overarching dimensions.

**Table 1:** 110 food parenting concepts grouped into 17 clusters based on qualitative cluster assignment informed by published Food Parenting Framework [11] of expert’s sorting (corresponds with Fig. 2)

Concept Number	Food Parenting Practice Concept	Hierarchal Analysis Point MapCluster Assignment^a^	Qualitative Content Map Cluster Assignment	Definition
Control				
54	I restrict my child’s food intake to control his/her weight.	3	A: Restriction for Weight	Due to concern for child’s weight, parent restricts access to or discourages consumption of certain foods, large portions, multiple servings, or frequent snacks.
68	I tell my child to avoid certain food or drinks as they can make him/her fat.	1		
22	I use food to soothe my child.	2	B: Using Food to Control Negative Emotions	“Parent uses food to manage or calm the child when he/she is upset, fussy, angry, hurt, or bored.” [11]
23	I give my child food to keep him/her occupied.	2		
17	I reward my child with something tasty (e.g. dessert) as a way to get him/her to eat [food/healthy food/all his/her dinner].	3	C: Threats and Bribes	Parent threatens to take or takes something away for misbehavior or promises/offers something to the child to coerce them into desired behavior. Threats and bribes related to food include those used to manage child’s general behavior by using food as reward or threat; or using threats or bribes to influence the child’s eating behaviors. (modified from Vaughn et al.) [11]
18	I tell my child that I will take away privileges (e.g., screen time) if s/he does not eat (./healthy food type).	3		
19	I punish my child (e.g., send away from table, spank) if s/he does not want to finish his/her plate, taste a food, or eat fruit or vegetables.	3		
20	I promise my child [unhealthy food] as a reward for good behavior.	3		
24	I scold or show disapproval when my child eats too much.	3		
25	I show disapproval by arguing with or yelling at my child for not eating [healthy food].	3		
73	I use scare tactics to discourage my child from eating unhealthy foods.	4		
88	I use threats to get my child to eat.	4		
89	I make my child feel guilty when s/he doesn’t eat vegetables or finish his/her meal.	3		
105	I withhold dessert as a consequence for bad behavior.	3		
106	I discipline my child if s/he consumes an unhealthy food/drinks without my permission.	4		
16	I make sure my child eats [all their dinner/all their fruits/vegetables] before s/he can have dessert.	4	D: Pressure to Eat	Parent is forceful or demanding in order to push the child to eat food, when child is either not interested in eating, not hungry, or does not want to eat or taste the food that is served during a meal or snack. The parent does not take into consideration the child’s current hunger or satiety, nor the child’s food preferences. (modified from Vaughn et al.) [11]
42	I beg my child to eat (./at least something from his/her plate).	4		
51	I make my child eat all the food on his/her plate.	4		
52	I get my child to eat more vegetables, even if s/he says “I’m not hungry.”	4		
53	I insist/force my child to “try one bite” or taste a [food/healthy food].	4		
21	I trick my child into eating [healthy food] by mixing it with other food or disguising it.	5	E: Intrusive Control	Parent dictates how and what the child should eat. Parent tells their child what to do and expects their child to comply without question. (NEW)
37	I tell my child to eat [healthy food] or not eat [unhealthy food/drinks] but do not follow this myself.	5		
39	I decide what my child should eat (./at meals/snacks).	6		
40	I don’t allow my child to eat more than I think s/he should.	5		
41	I make my child eat [healthy food] every day.	5		
55	If my child eats more than usual at one meal, I try to restrict his/her eating at the next meal.	5		
72	I make my child eat his/her fruit and vegetables first at mealtimes or snacks.	5		
87	I criticize my child about the food s/he eats.	4		
97	I decide when my child eats his/her meals and snacks.	5		
Structure				
49	I have to strongly encourage my child to eat foods that are good for him/her.	1	F: Prompt to Eat	Parent suggests to the child or prompts the child to eat food without being forceful and without consequence. There is no focus on eating beyond satiety. There is an emphasis on promoting to eat nutritious food. (NEW)
50	I encourage my child to eat all the food on his/her plate.	1		
98	I try to convince my child to eat fruit or vegetables instead of cake or candy.	1		
100	I tell my child to eat fruit and vegetables.	2		
38	I monitor or keep track of the [healthy/unhealthy food/drinks] my child eats/drinks.	7	G: Rules and Limits	Parent has and makes known expectations, guidelines, or boundaries for how much or what kind of foods the child eats, maintains the timing or routine of meals, or promotes a certain order in which foods are eaten. The parent can monitor whether the child sticks to the rules. (modified from Vaughn et al.) [11]
44	I do not allow my child to eat or drink an hour before meals or after a certain hour of the day.	7		
45	I ask others not to give my child unhealthy food (candy, sweets, salty snacks).	7		
56	I limit or do not allow my child to eat/drink certain [unhealthy food/drinks].	6		
59	I insist my child eat meals/snacks at the table.	8		
74	If I allow my child an unhealthy meal/snack the next meal snack must be healthy.	6		
99	If my child eats junk food, s/he must also include something healthy.	6		
11	I allow my child to have whatever sweets and snacks s/he chooses at social occasions or to celebrate an achievement.	10	H: Permissive Feeding	“Parent allows child complete control of their eating, including timing and frequency of meals and snacks, and amount and type of foods eaten.” [11] Parent does not impose limits and will provide different foods for the child from what the rest of the family eats based on the child’s preferences and whims. Parent facilitates less nutritious food selection by keeping those in the home or taking the child place those foods are served.
14	I serve dessert to my child if s/he is no longer hungry for her/his main dish but is willing to eat dessert.	8		
61	I take my child to eat at fast food places.	10		
79	I serve/offer unhealthy foods [at meals/snacks/for dessert].	9		
91	I offer my child seconds.	8		
96	I eat/drink unhealthy foods/drinks with my child.	10		
3	I give my child money to buy food (snacks, treats, or meals).	10		
7	I allow my child to eat whenever s/he is hungry or shows signs of hunger.	10		
8	I allow my child to buy [unhealthy food type] if s/he wants it as a snack or meal.	9		
10	I serve what my child demands at meals.	9		
12	I make or allow my child to make something else if s/he does not like what is served.	9		
15	I give into my child’s food demands (./after saying no)	9		
69	I allow my child to eat unhealthy when we are away from home (e.g., doing errands, driving to practices).	9		
80	I allow my child to have seconds if s/he finishes foods from his/her plate at dinner.	8		
81	I let my child eat unhealthy food whenever s/he wants.	9		
82	I let my child substitute a food s/he does not like for one s/he likes.	10		
107	I allow my child to skip meals.	8		
110	I allow my child to eat unhealthy when we are on vacation.	9		
27	I hide or intentionally keep less [healthful food/drinks] out of my child’s reach.	27	I: Food Accessibility/Availability	The amount and types of foods that a parent brings into the home; or how readily accessible the parent makes healthy and unhealthy foods in the home. Accessibility includes making foods ready and easy to eat, such as washing, cutting up and making food easy to see and reach for the child. (Modified from Vaughn et al.) [11]
28	I keep or have ready to eat fruits and vegetables in the fridge for my child to eat (e.g., pre-cut, clean).	10		
29	I avoid having [unhealthy food/drinks] available at home.	10		
75	I make sure that I have healthy foods in the house (./that my child likes).	10		
76	I have unhealthy foods in the house (./that my child likes).	10		
31	I include [healthy food] in my child’s lunch/snacks/meals (./that s/he likes).	10	J: Food Preparation	The planning, preparation and cooking methods that a parent employs when providing or serving meals and snacks, which may impact the healthfulness of the foods served. (Modified from Vaughn et al.) [11]
32	I balance all food groups in my child’s meals.	10		
43	I plan and prepare my child’s meals/school lunches (./from scratch).	10		
93	I prepare food in a low-fat or healthy way for my family.	11		
94	I use pre-packaged, convenience food for meals.	10		
26	I show enthusiasm about eating healthy foods.	14	K: Modeling	Eating specific behaviors the parent engages in themselves in front of child that may entice their child to emulate his/her eating behaviors, food choices, or amounts of food. It can either be in regards to nutritious foods or less nutritious foods. It is distinct from creating opportunities to role model eating behaviors such as having a family meal together.
34	I eat/drink [healthy food/drinks] in front of my child (./even if they are not my favorite).	11		
35	I avoid eating/drinking [unhealthy food/drinks] in front of my child.	11		
95	I take a second helping of food at dinner in front of my child.	11		
30	I serve [healthy food] multiple times and in different ways to encourage my child to develop a taste for it.	12	L: Exposure to Variety/Selection	Parent exposes the child to nutritious and/or different food on a regular basis, includes variety of ways to prepare or eat nutritious food, and may allow choice for the child. (NEW)
71	I expose my child to a variety of fruits and vegetables (./since s/he was little).	12		
78	I serve/offer [healthy food type] (./each day, for snacks, for a side-dish, for breakfast/ for dinner/for dessert)	12		
92	To ensure my child eats a particular food (e.g., vegetables), I serve it with food my child likes.	12		
108	I suggest places to eat out that have healthy selections for my child.	12		
58	I try to minimize distractions during mealtimes (e.g., watching TV, answering phone calls, texting, playing with toys).	12	M: Meal Routines	“Parent implements consistency and predictability around meals and snacks with regard to their location, timing, presence of family members, conversational tone, and presence/absence of distractions.” [11]
60	I make sure my family eats together as often as possible.	11		
4	I give my child small portions to get him/her to eat a particular food or new foods.	13	N: Redirection and Negotiation	“Parent engages with child to come to an agreement about what or how much the child will eat. Negotiation allows for resolution of different opinions between parent and child by finding an acceptable compromise.” [11] Parent uses tactics to take the child’s mind off of certain foods or drinks, provides them with alternatives, or shares food to decrease portion size. The tactics are not forceful and there are no consequences if not successful.
5	I offer/provide my child healthy options when s/he asks for unhealthy food or treats.	13		
6	I negotiate with my child about how much unhealthy or healthy food s/he eats or drinks.	15		
77	I encourage my child to eat [vegetables] by playing games with my child at meals times or by challenging him/her to eat it.	13		
102	I encourage my child to drink water when s/he feels hungry.	13		
103	To discourage my child from eating a particular food, I give him/her something else to do.	13		
104	I encourage my child to control his/her intake of unhealthy food/drinks by sharing it.	13		
Autonomy Support				
1	I take into account the [healthy food/drinks] my child likes when shopping for food or preparing meals.	12	O: Child involvement	Parent acknowledges the child is an independent individual and takes into consideration the preferences and wants of the child by actively involving the child during meal planning, grocery shopping, meal preparation, or mealtime, with the goal to motivate more nutritious intake (modified from Vaughn et al.) [11]
2	I allow my child to serve him/herself and decide how much food s/he eats.	12		
9	I let my child have a lot of say in what is eaten or prepared for meals.	14		
13	I let my child season the vegetables, such as adding ketchup or cheese sauce, to make them taste better.	7		
57	I talk to my child during meals.	14		
62	I ask my child to suggest how s/he can eat more healthy food.	15		
83	If my child does not want to taste a food, I do not try to make him/her eat it.	7		
84	I let my child prepare his/her lunch/snacks.	14		
85	I involve my child in meal and snack preparation.	14		
86	I let my child choose fruits and vegetables while grocery shopping.	14		
33	I encourage my child to eat [healthy food] by making the food interesting (e.g., cutting into shapes, preparing it in a variety of ways, or seasoning it).	14	P: Encourage Health Eating	Non-directive methods to suggest that the child try or eat a healthy food, but is not forceful and does not have consequences associated with child not following through. These non-directive methods include gentle verbal cues or reminders, non-verbal methods by making food more appealing or interesting for child. It also includes promoting self-regulation of intake by children to not eat beyond satiety. (Modified from Vaughn et al.) [11]
36	I tell my child how much I like a food to encourage him/her to eat it.	15		
46	I encourage my child to eat/drink/try [healthy food] (./but do not force him/her to do so)	15		
47	I encourage my child to eat/drink [healthy food/drinks] instead of or before [unhealthy food/drinks].	15		
48	I tell my child that his/her friends/sibling(s)/favorite characters like the [healthy food] as a way to encourage him/her to eat it.	15		
70	I remind/encourage my child to stop eating or to not take more food when s/he feels full.	15		
90	I praise my child for eating healthy food or fruit and vegetables.	16		
101	I help my child set a goal to eat more fruit and vegetables.	15		
63	I persuade my child to eat healthy food by explaining why it’s important (e.g., you will feel better, good for you, you’ll grow big and strong, do better at school).	16	Q: Education/Reasoning	Explanations given by parent to child to educate the child about foods’ nutritional qualities, such as the benefits of eating healthy foods or the consequences of eating unhealthy ones. Parent uses logic or explanations to persuade the child to change his or her eating behavior (modified form Vaughn et al.) [11]
64	I teach my child that certain food/drinks should only be consumed in moderation.	16		
65	I tell my child that certain food or drinks are not good for his/her health or teeth.	16		
66	I use mealtimes to teach my child about healthy eating.	16		
67	I teach my child about healthy eating by reading food labels and playing educational games.	16		
109	I give my child ideas on how to eat healthier (e.g., eating more fruits and vegetables).	16		

^a^This refers to the cluster the item was assigned based on the original 16-cluster solution identified by the cluster analysis. This is visually depicted in Fig. 2 as the gray shadow clusters and illustrates how many subjective changes were made to generate the proposed 17 cluster solution

Most of the food parenting practice constructs under each dimension defined by Vaughn and colleagues’ content map [11] appear to also cluster on the comprehensive concept map (Fig. 2). All four Coercive Control constructs identified on the content map were spatially close and therefore labeled to belong to Control on the comprehensive concept map: Restriction (A), Using Food to Control Negative Emotions (B), Threats & Bribes (C), and Pressure to Eat (D). One notable difference in our solution was the construct of Restriction (A) was specific for controlling weight, whereas in the content map Restriction was a more general concept. Another difference was the addition of a new construct under Control, termed Intrusive Control (E). This construct included demanding and directive concepts where the parent dictated what and how much the child should eat. These demanding and directive concepts were distinct from pressuring the child to eat more, as seen in Pressure to Eat (D), and from the guidelines and boundaries that parents set, found in the Rules and Limits construct (G) under the Structure dimension. Intrusive Control was therefore made into a new construct. It was included in the Control domain because the focus was on parents dictating to the child without child input.

The proposed content map [11] identified nine constructs under Structure, of which six were identified in the comprehensive concept map solution: Rules and Limits (G), Food Availability and Accessibility (I), Food Preparation (J), Modeling (K), Meal Routines (M), and Permissive (H) (or “unstructured practices” as termed by Vaughn et al. [11].) The Availability and Accessibility construct was separated into two constructs by Vaughn et al. [11], however, the comprehensive concept map solution collapsed it into one construct. In the comprehensive concept map, there was a lack of a distinct Monitoring category in the Structure dimension as defined by the published content map, which may be due to the multiple published items on monitoring being condensed down into one monitoring concept (# 38) for this sorting task. In the solution presented here it falls into the Rules and Limit construct, but future studies will need to assess whether it should be a separate construct in the Structure dimension.

Different from Vaughn’s et al. content map, three new categories were identified in the comprehensive concept map under the Structure dimension: Prompt to Eat (F), Exposure to a Variety/Selection (L) and Redirection & Negotiation (N). Upon review of the solution, some experts identified similarities of the concepts clustered in Prompt to Eat to concepts clustered under Pressure to Eat. However, the two clusters were spatially separate from each other on the map. Therefore, Prompt to Eat was identified as a distinct construct from Pressure to Eat and reflected more gentle reminders for a child to eat, as opposed to pushing the child to eat beyond satiety as seen in Pressure to eat. This difference suggests that the Experts may distinguish varying degrees of how parents remind or push their child to eat and some Experts felt that Prompt to Eat was a form of Structure instead of Control. Exposure to a Variety/Selection was not identified by the published content map but concepts that clustered into this construct spatially fell into the Structure dimension on the comprehensive concept map. On face validity, these concepts may be an extension of availability, but the concepts were spatially separate from the Availability and Accessibility construct on the map and therefore made into a new construct. Redirection & Negotiation was also not a construct in the content map proposed by Vaughn et al., but does have some overlap with the content map’s Limited/Guided Choices (which was not present in the solution presented in Fig. 2). Future work will need to explore the overlap and differences between these two constructs.

The Autonomy Promotion dimension had the most differences between the comprehensive concept map and the previously proposed content map [11]. Similar to the proposed framework, Child Involvement (O) was a distinct construct under Autonomy Promotion. However, the two proposed constructs of Praise and Encouragement were combined into a single Encourage Healthy Eating (P) construct, while the two proposed Nutrition Education and Reasoning constructs were combined into a single Education/Reasoning (Q) construct. Lastly, the proposed construct Negotiation, which Vaughn et al. suggested belonged in Autonomy Promotion [11] was instead collapsed with Redirection in the Structure dimension (Fig. 2).

Five concepts (13, 26, 27, 83, and 87- Fig. 2) were grouped with clusters spatially removed from their closest cluster on the point map, because the research team deemed they fit better conceptually. Food parenting practice concepts 13 (I let my child season the vegetables, such as adding ketchup or cheese sauce, to make them taste better) and 83 (If my child does not want to taste a food, I do not try to make him/her eat it), were spatially closest to Rules and Limits and Permissive Feeding, respectively. However, the team proposed both concepts fit better into Child Involvement, which includes concepts that allow the parent to consider their child as an individual when motivating them to eat more nutritious foods. Concepts 26 (I show enthusiasm about eating healthy foods.) was spatially within Child involvement (P), but was moved into Modeling (M), to capture the concept of enthusiastic modeling [17], as per recommendation of Experts. Concept 27 (I hide or intentionally keep less [healthful food/drinks] out of my child’s reach) has sometimes been classified as a form of covert control, but several experts felt it better fit into J: Availability/Accessibility. Concept 87 (I criticize my child about the food s/he eats.), was initially grouped with Pressure to Eat concepts, but it did not promote eating more food like the other concepts in Pressure to Eat. It was therefore moved to the adjacent new construct Intrusive Control which focused on directive and intrusive parental control of their child.

### The parsimonious conceptual framework of food parenting practices

The parsimonious solution was derived from the 4-cluster statistical solution which expanded to a 7-cluster solution after it was subjectively reviewed and endorsed as part of the consensus process. The final model can be found in Additional file 1: Figure A (online) with construct names, definitions and corresponding concepts found in Additional file 1: Table A (online). The subjective separation of clusters was performed because the increased number into 5, 6 or 7 clusters resulting from the hierarchical cluster solution did not fit based on face validity. Instead, subjective modifications to the 4-cluster solution were based on the research team’s current understanding of the published literature. The first modification was due to one of the 4 clusters containing unique concepts for different forms of coercive control (e.g. punitive restriction and pressure to eat). Prior research suggests that parents use pressure to eat more with picky eaters or underweight children, and it has been associated with lower weight status among children in several cross-sectional and longitudinal studies [18–21]. On the other hand, restriction has been more commonly associated with higher child weight status in cross-sectional and longitudinal studies and may be a response to children who are heavier or are more food responsive [18–21]. These divergent outcomes associated with pressure to eat and restriction suggested these two constructs may be conceptually different and should be measured independently of each other. Therefore, all the concepts in this group that reflected pushing children to eat more (whether coercive or not) were moved to the Pressure to Eat construct (Cluster 1). All the concepts that reflected the use of punishment or coercion to restrict the amount that children could eat remained in the Restriction construct (Cluster 2).

The next modification involved a large cluster that emerged from the 4 cluster solution which contained concepts related to parental Rules and Expectations (Cluster 4), along with two concepts (concepts 22 and 23) that theoretically did not belong with the others. These two concepts on the border of the cluster were more consistent with the idea of Emotional Feeding, first identified by Wardle et al. [22], and were therefore separated into a different construct named Emotional Feeding (Cluster 3).

The statistically derived 4-cluster solution included one large cluster that combined concepts for creating structure for a child with indulgent food parenting practices. Indulgent feeding style has consistently been associated with higher child weight status in cross-sectional [23] and recently in a longitudinal study [24]. However, structure is believed to be protective from excessive weight gain among children and for ensuring adequate consumption and growth for children with low weight status. It was considered whether these constructs are at the opposite ends of one spectrum, but we believed it is possible that parents can be indulgent with or without structure. The last modification therefore involved separating these two constructs into Indulgence (Cluster 5) and Structure (Cluster 6). The final cluster identified in the statistically derived 4-cluster solution contained strategies that involved parental Active Encouragement for Nutritious Eating by their child, and remained intact (Cluster 7). Based on expert input on the solution, items that may require further evaluation for fitting within each construct are identified for future studies in Additional file 1. A comparison of the two solutions can be found in Additional file 1: Figure B (online).

## Discussion

An international expert panel of researchers involved in food parenting practices research helped guide the development of a new Concept Map for Food Parenting Practices. Both of the final two Food Parenting Practices Concept Maps presented here were derived from a MDS point map of the spatial relationships of 110 food parenting concepts based on the sorting task of 28 food parenting experts from around the world. One of the concept maps (Fig. 2, Table 1) is a subjective clustering of the food parenting practice concepts point map that retains a more comprehensive structure and was informed by the developmental psychology literature cited by Vaughn et al. [11] This comprehensive concept map should allow researchers to evaluate the impact of each of the proposed food parenting practice constructs on child outcomes, how parents use these practices in combination [25, 26], and whether child characteristics moderate the impact of each construct. This detailed solution will also allow researchers to select specific constructs when testing hypothesis or developing or evaluating interventions. The other concept map (Additional file 1: Figure A and Table A) was informed by the hierarchical cluster analysis solution and took a parsimonious approach as we aimed at identifying fewer clusters or constructs. This latter approach may help scientists enhance their measurement of food parenting practice by reducing the burden of measurement, while assessing more global and potentially predictive constructs. After they were given the opportunity to review both solutions, over half of the experts preferred the comprehensive solution. They felt this model allows for a better distinction of which constructs are most predictive of child behavior and health outcomes and have a greater impact to move this area of research forward.

Both the comprehensive and parsimonious solutions required subjective modifications to the statistically derived cluster solutions from the MDS point map. The difficulty in interpreting any of the hierarchical cluster analysis solutions without modifications, suggest that there was not great consensus among these experts for how to conceptualize a framework for food parenting practice, despite being provided with a published content map that several of the investigators and experts in this study helped develop. Of note, the cluster analysis of the MDS solution of only those experts that participated in the development of the published content map [11] and this sorting task was also explored, with no clearer solution apparent. One expert suggested that the comprehensive solution may be sub-factors within the more global parsimonious solution. Unfortunately, the current solutions do not fully support this as illustrated in Additional file 1, where there is not always clear overlap between the constructs defined in the two solutions. It is possible that future studies can help further refine both solutions such that the relationships between the two can be better delineated.

In this study, an international group of experts helped develop a concept map for food parenting practices using a systematic approach to identify the food parenting practice concepts, by allowing them to sort the concepts into categories and interpret their sorting using statistical analysis. This is distinct from the approach taken to develop Vaughn et al.’s content map [11], for which an overlapping group of experts were asked to collaboratively propose a framework for food parenting practices based on their own research and review of the literature. This published content map currently lacks validation. While the intent of this study was not to validate the published content map, the team felt it was important to allow the experts access to it. Since the food parenting content map was not published at the time the experts were asked to complete the sorting task, it was provided to allow them to use all possible resources. They were instructed to use the framework only if it worked with their own conceptual approach to the sorting task. It is not known how many elected to do so.

The work presented here was based on an item bank developed from published instruments of food parenting practice instruments systematically identified in 2013 [4]. Since that time, additional studies have been published that adapted or tested the psychometrics of food parenting practice scales already included in the item bank, to new populations [27–31]. In addition, several important new instruments of food parenting practices have been published that could not be included in the item bank to inform the Experts’ tasks. These include the Parental Feeding Practices (PFQ) scale for Mexican American families [32], the Vegetable Parenting Practice scale [33], Feeding Practices and Structure Questionnaire (FPSQ-28) [34], and the Structure and Control in Parent Feeding (SCPF) [35]. However, several of these instruments were developed based on previously published scales and had much overlap with items already included in the item bank and would likely integrate with the concepts we identified. The extent to which these newer items fit within our existing concepts will be tested empirically in the future.

The goal of this study is ultimately to improve the measurement of food parenting practices to allow for a more standardized assessment of food parenting practice constructs and better comparisons of results across studies. The team is currently iteratively developing items to cover the constructs presented in the comprehensive food parenting practice solution using existing or modified items from published scales. Future work will include testing the resulting questionnaire in English with parents via cognitive interviews, and then assessing parents’ use of the food parenting practices in a large cross-sectional study for classical test theory and advanced psychometric analysis (e.g. item response modeling). This will allow psychometric testing of the proposed comprehensive model. If the comprehensive model is a poor fit which cannot be improved with minor alterations, the parsimonious model will be tested instead. Ultimately, the goal is to create a calibrated item bank that can be used for computer assisted testing in observation and interventions studies. The psychometric analysis will help achieve this by assessing whether items are stable across participant characteristics (e.g. income and education) via differential item functioning within a Canadian sample. Future work will need to assess the stability of items across different cultural racial and ethnic groups and in different languages. The improvements in measuring food parenting practices will hopefully result in more consistent use of instruments across studies and better understanding of the impacts of food parenting practices on child outcomes, and whether child characteristics or behaviors moderate these findings.

### Strengths and limitations

This study has several strengths including a systematic approach to identifying food parenting practice concepts, engaging an international sample of experts in food parenting and child feeding in a sorting task, and using both quantitative and subjective approaches to interpret the resulting point map solution of food parenting practices. Limitations of this methodology should also be acknowledged. The item bank is based on instruments published before March 2013, and therefore does not include instruments developed after this time. Experts varied by how much they relied on the previously published content map [11] to inform their own sorting, which may have influenced the final analysis. The cluster analysis derived solution of food parenting practice clusters also suggested there was variability in how the Experts operationalized each construct. Another limitation in measuring food parenting practice concepts is they are typically operationalized as unidirectional behaviors of parents aimed at their child. This may imply the assumption that we can fully understand the impact of food parenting practice on child dietary behavior by applying the Concept Map in observational and intervention studies. It should be acknowledged that food parenting practice is embedded in a more complex family context, including the back-and-forth interactions between a parent and a child, as well as sibling and marital relationships, with reciprocal influences between all family members. Thus, it is likely that food parenting practices can be best understood in the context of interactions within the family as a whole.

## Conclusion

In summary, the comprehensive food parenting practice concept map derived from the experts’ sorting of food parenting practice concepts provides a conceptual map and the roadmap for the selecting and developing of items for each construct. These will in turn be tested to eventually develop a calibrated item bank of food parenting practices, which will help standardize the measurement of food parenting practice in future observational and intervention studies.

## Additional file

Additional file 1: Parsimonious solution of food parenitng practices. (DOCX 356 kb)

## Abbreviations

FPP: Food Parenting Practices
IRM: Item Response Modeling
ISBNPA: International Society of Behavioral Nutrition and Physical Activity
MDS: Multidimensional Scaling
NIH: National Institute of Health
