# Supplementary material for: Food parenting practices for 5 to 12 year old children: a concept map analysis of parenting and nutrition experts input
Source: Int J Behav Nutr Phys Act. 2017 Sep 11;14:122. doi: 10.1186/s12966-017-0572-1 (PMC5594481; doi:10.1186/s12966-017-0572-1)

| **Additional file 1.** Parsimonious Solution of 110 food parenting practices grouped into 7 clusters informed by hierarchical cluster analysis solution (corresponds with appendix Figure A) | | | |
| --- | --- | --- | --- |
| **Concept Number** | **Food Parenting Practice Concept** | **Seven Cluster Solution Construct Assignment** | **Definition** |
| 16 | I make sure my child eats [all their dinner / all their fruits/vegetables] before s/he can have dessert. | 1. Pressure to Eat | “Parent insists, coerces, demands, or physically struggles with the child in order to get the child to eat more food.”^11^ |
| 42 | I beg my child to eat (./at least something from his/her plate). |  |  |
| 51 | I make my child eat all the food on his/her plate. |  |  |
| 52 | I get my child to eat more vegetables, even if s/he says "I'm not hungry." |  |  |
| 53 | I insist/force my child to "try one bite" or taste a [food/healthy food]. |  |  |
| 89 | I make my child feel guilty when s/he doesn’t eat vegetables or finish his/her meal. |  |  |
| 18 | I tell my child that I will take away privileges (e.g., screen time) if s/he not does eat (./healthy food type). |  |  |
| 19 | I punish my child (e.g., send away from table, spank) if s/he does not want to finish his/her plate, taste a food, or eat fruit or vegetables. |  |  |
| 88 | I use threats to get my child to eat. |  |  |
| 17 | I reward my child with something tasty (e.g. dessert) as a way to get him/her to eat [food / healthy food/ all his/her dinner]. |  |  |
| 25 | I show disapproval by arguing with or yelling at my child for not eating [healthy food]. |  |  |
| 87 | I criticize my child about the food s/he eats. | 1. Restriction | Parent is dominant and intrusive, and exerts control in relation to their child’s eating behaviors to enforce strict limits and restrictions on child. (modified from Vaughn et al. 2015)^11^ |
| 54 | I restrict my child's food intake to control his/her weight. |  |  |
| 73 | I use scare tactics to discourage my child from eating unhealthy foods. |  |  |
| 20 | I promise my child [unhealthy food] as a reward for good behavior.* |  |  |
| 24 | I scold or show disapproval when my child eats too much. |  |  |
| 105 | I withhold dessert as a consequence for bad behavior. |  |  |
| 106 | I discipline my child if s/he consumes an unhealthy food/drinks without my permission. |  |  |
| 22 | I use food to soothe my child. | 1. Emotional Feeding | “Parent uses food to manage or calm the child when he/she is upset, fussy, angry, hurt or bored.” ^11^ |
| 23 | I give my child food to keep him/her occupied. |  |  |
| 5 | I offer/provide my child healthy options when s/he asks for unhealthy food or treats.* | 1. Rules and Expectations | Parent has and makes known expectations, guidelines, or boundaries for how much or what kind of foods the child eats, maintain the timing or routine of meals, or promote a certain order in which foods are eaten. It includes parent reminding child of expectations and/or rules. The rules are there to be followed and the parent can monitor whether the child sticks to the rules (modified from Vaughn et al.) ^11^ |
| 21 | I trick my child into eating [healthy food] by mixing it with other food or disguising it. |  |  |
| 38 | I monitor or keep track of the [healthy/unhealthy food/drinks] my child eats/drinks. |  |  |
| 49 | I have to strongly encourage my child to eat foods that are good for him/her.* |  |  |
| 68 | I tell my child to avoid certain food or drinks as they can make him/her fat.* |  |  |
| 77 | I encourage my child to eat [vegetables] by playing games with my child at meals times or by challenging him/her to eat it. |  |  |
| 98 | I try to convince my child to eat fruit or vegetables instead of cake or candy. |  |  |
| 100 | I tell my child to eat fruit and vegetables.* |  |  |
| 103 | To discourage my child from eating a particular food, I give him/her something else to do. |  |  |
| 50 | I encourage my child to eat all the food on his/her plate. |  |  |
| 4 | I give my child small portions to get him/her to eat a particular food or new foods. |  |  |
| 104 | I encourage my child to control his/her intake of unhealthy food/drinks by sharing it. |  |  |
| 40 | I don't allow my child to eat more than I think s/he should. |  |  |
| 55 | If my child eats more than usual at one meal, I try to restrict his/her eating at the next meal. |  |  |
| 102 | I encourage my child to drink water when s/he feels hungry.* |  |  |
| 37 | I tell my child to eat [healthy food] or not eat [unhealthy food/drinks] but do not follow this myself. |  |  |
| 39 | I decide what my child should eat (./at meals/snacks). |  |  |
| 41 | I make my child eat [healthy food] everyday.* |  |  |
| 45 | I ask others not to give my child unhealthy food (candy, sweets, salty snacks). |  |  |
| 56 | I limit or do not allow my child to eat/drink certain [unhealthy food/drinks]. |  |  |
| 74 | If I allow my child an unhealthy meal/snack the next meal snack must be healthy. |  |  |
| 99 | If my child eats junk food, s/he must also include something healthy. |  |  |
| 44 | I do not allow my child to eat or drink an hour before meals or after a certain hour of the day. |  |  |
| 97 | I decide when my child eats his/her meals and snacks. |  |  |
| 72 | I make my child eat his/her fruit and vegetables first at mealtimes or snacks. |  |  |
| 13 | I let my child season the vegetables, such as adding ketchup or cheese sauce, to make them taste better. |  |  |
| 83 | If my child does not want to taste a food, I do not try to make him/her eat it. |  |  |
|  |  |  |  |
| 2 | I allow my child to serve him/herself and decide how much food s/he eats.* | 1. Indulgence | Parent gives in to child’s demands and preferences by coddling to the child’s desires, in a way that prohibits the child from learning to accept and like new foods. Instead it promotes intake of food that are less nutritious. |
| 3 | I give my child money to buy food (snacks, treats, or meals). |  |  |
| 8 | I allow my child to buy [unhealthy food type] if s/he wants it as a snack or meal. |  |  |
| 10 | I serve what my child demands at meals. |  |  |
| 11 | I allow my child to have whatever sweets and snacks s/he chooses at social occasions or to celebrate an achievement. |  |  |
| 12 | I make or allow my child to make something else if s/he does not like what is served. |  |  |
| 14 | I serve dessert to my child if s/he is no longer hungry for her/his main dish but is willing to eat dessert. |  |  |
| 15 | I give into my child's food demands (./after saying no) |  |  |
| 69 | I allow my child to eat unhealthy when we are away from home (e.g., doing errands, driving to practices). |  |  |
| 79 | I serve/offer unhealthy foods [at meals/snacks/for dessert]. |  |  |
| 80 | I allow my child to have seconds if s/he finishes foods from his/her plate at dinner. |  |  |
| 81 | I let my child eat unhealthy food whenever s/he wants. |  |  |
| 91 | I offer my child seconds. |  |  |
| 107 | I allow my child to skip meals. |  |  |
| 110 | I allow my child to eat unhealthy when we are on vacation. |  |  |
| 82 | I let my child substitute a food s/he does not like for one s/he likes. |  |  |
| 61 | I take my child to eat at fast food places. |  |  |
| 76 | I have unhealthy foods in the house (./that my child likes).* |  |  |
| 96 | I eat/drink unhealthy foods/drinks with my child. |  |  |
| 7 | I allow my child to eat whenever s/he is hungry or shows signs of hunger. |  |  |
|  |  |  |  |
| 27 | I hide or intentionally keep less [healthful food/drinks] out of my child's reach. | 1. Structure | Proactive, environmental changes or manipulations to promote nutritious and appropriate intake and facilitate child’s competence in eating nutritious food. It includes making nutritious foods available and accessible, serving nutritious foods, exposing children to a variety of choices, role modeling, and promoting family meals. |
| 28 | I keep or have ready to eat fruits and vegetables in the fridge for my child to eat (e.g., pre-cut, clean). |  |  |
| 29 | I avoid having [unhealthy food/drinks] available at home. |  |  |
| 75 | I make sure that I have healthy foods in the house (./that my child likes). |  |  |
| 108 | I suggest places to eat out that have healthy selections for my child. |  |  |
| 30 | I serve [healthy food] multiple times and in different ways to encourage my child to develop a taste for it. |  |  |
| 71 | I expose my child to a variety of fruits and vegetables (./since s/he was little). |  |  |
| 92 | To ensure my child eats a particular food (e.g., vegetables), I serve it with food my child likes. |  |  |
| 34 | I eat/drink [healthy food/drinks] in front of my child (./even if they are not my favorite). |  |  |
| 35 | I avoid eating/drinking [unhealthy food/drinks] in front of my child. |  |  |
| 95 | I take a second helping of food at dinner in front of my child.* |  |  |
| 58 | I try to minimize distractions during mealtimes (e.g., watching TV, answering phone calls, texting, playing with toys). |  |  |
| 60 | I make sure my family eats together as often as possible. |  |  |
| 31 | I include [healthy food] in my child's lunch/snacks/meals (./that s/he likes). |  |  |
| 32 | I balance all food groups in my child's meals. |  |  |
| 43 | I plan and prepare my child's meals/school lunches (./from scratch). |  |  |
| 78 | I serve/offer [healthy food type] (./each day, for snacks, for a side-dish, for breakfast/ for dinner/for dessert) |  |  |
| 93 | I prepare food in a low-fat or healthy way for my family. |  |  |
| 94 | I use pre-packaged, convenience food for meals.* |  |  |
| 1 | I take into account the [healthy food/drinks] my child likes when shopping for food or preparing meals. |  |  |
| 59 | I insist my child eat meals/snacks at the table. |  |  |
|  |  |  |  |
| 9 | I let my child have a lot of say in what is eaten or prepared for meals. | 1. Active Encouragement of Nutritious Eating | Parents teach their child about healthy eating, reason with their child, uses nontangible positive reinforcement (e.g. praise and encouragement), and help their child get involved in decision making and preparation of foods. |
| 62 | I ask my child to suggest how s/he can eat more healthy food. |  |  |
| 84 | I let my child prepare his/her lunch/snacks.* |  |  |
| 85 | I involve my child in meal and snack preparation. |  |  |
| 86 | I let my child choose fruits and vegetables while grocery shopping. |  |  |
| 26 | I show enthusiasm about eating healthy foods. |  |  |
| 33 | I encourage my child to eat [healthy food] by making the food interesting (e.g., cutting into shapes, preparing it in a variety of ways, or seasoning it). |  |  |
| 36 | I tell my child how much I like a food to encourage him/her to eat it. |  |  |
| 46 | I encourage my child to eat/drink/try [healthy food] (./but do not force him/her to do so) |  |  |
| 48 | I tell my child that his/her friends/sibling(s)/favorite characters like the [healthy food] as a way to encourage him/her to eat it. |  |  |
| 90 | I praise my child for eating healthy food or fruit and vegetables. |  |  |
| 6 | I negotiate with my child about how much unhealthy or healthy food s/he eats or drinks. |  |  |
| 47 | I encourage my child to eat/drink [healthy food/drinks] instead of or before [unhealthy food/drinks]. |  |  |
| 63 | I persuade my child to eat healthy food by explaining why it's important (e.g., you will feel better, good for you, you'll grow big and strong, do better at school). |  |  |
| 64 | I teach my child that certain food/drinks should only be consumed in moderation. |  |  |
| 65 | I tell my child that certain food or drinks are not good for his/her health or teeth. |  |  |
| 66 | I use mealtimes to teach my child about healthy eating. |  |  |
| 67 | I teach my child about healthy eating by reading food labels and playing educational games. |  |  |
| 70 | I remind/encourage my child to stop eating or to not take more food when s/he feels full. |  |  |
| 101 | I help my child set a goal to eat more fruit and vegetables. |  |  |
| 109 | I give my child ideas on how to eat healthier (e.g., eating more fruits and vegetables). |  |  |
| 57 | I talk to my child during meals.* |  |  |
| *Item identified by one or more experts as not fitting current definition with a suggestion to move to another construct or deleting concept. | | | |


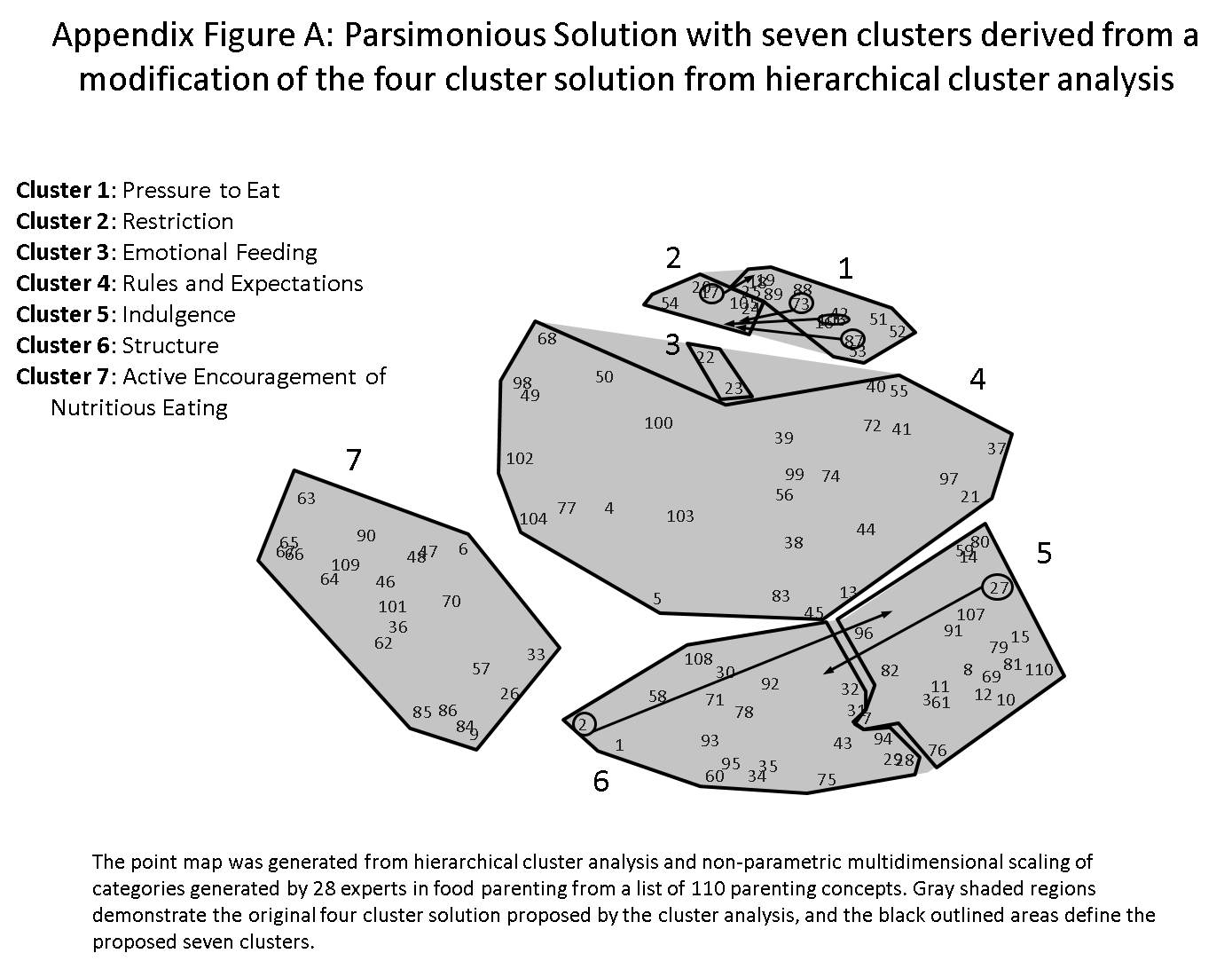


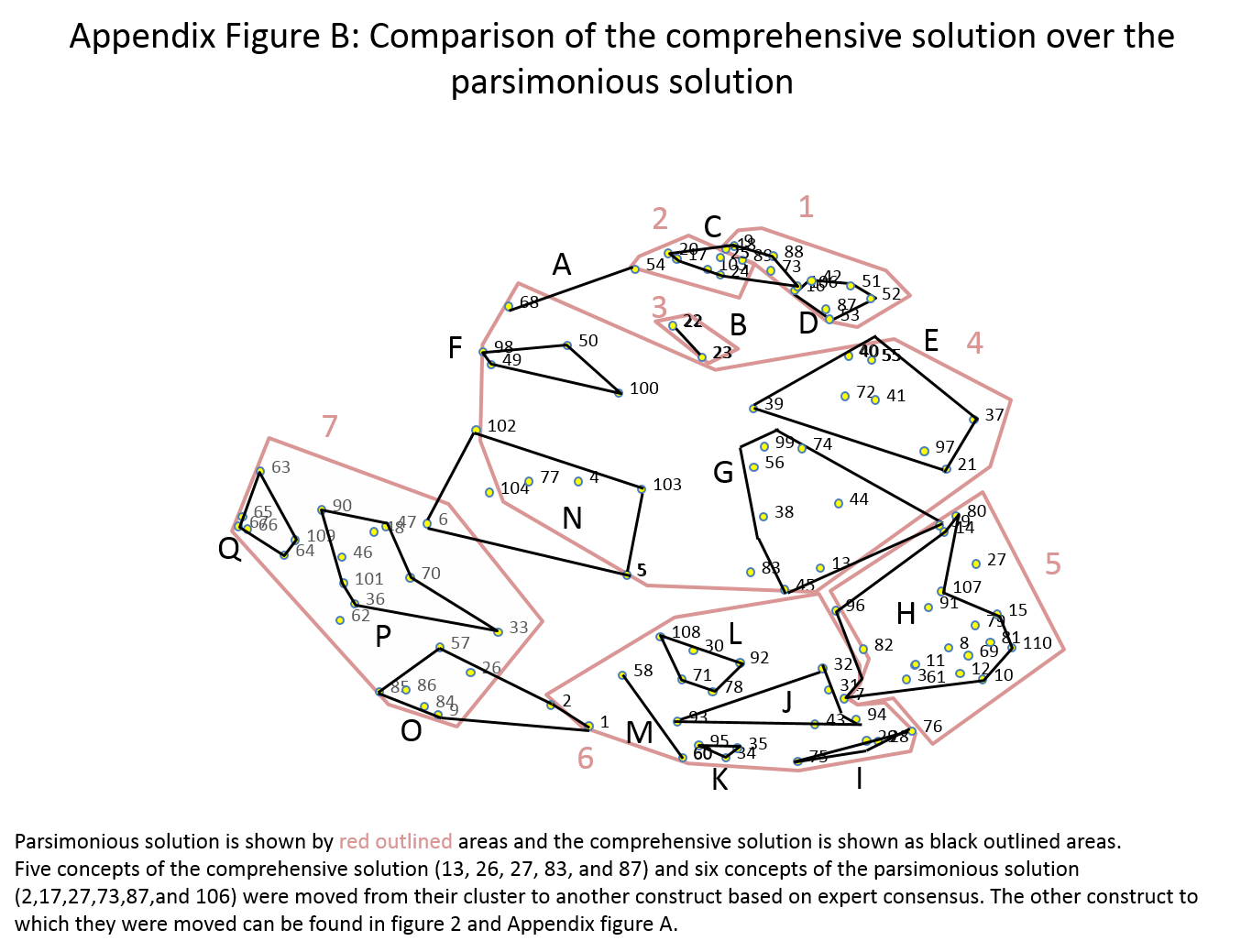

Supplement: Additional file 1: — Parsimonious solution of food parenitng practices. (DOCX 356 kb) [file 12966_2017_572_MOESM1_ESM.docx]
